# Time-resolved urinary proteomics reveals heme-associated oxidative stress responses in neonatal hypoxic-ischaemic encephalopathy

**DOI:** 10.1186/s40348-026-00222-7

**Published:** 2026-03-04

**Authors:** Magdalena Zasada, Maciej Suski, Marta Olszewska, Aleksandra Kowalik, Natalia Łapińska, Weronika Pogoda, Przemko Kwinta

**Affiliations:** 1 Department of Pediatrics, Jagiellonian University Medical College, University Children’s Hospital, Wielicka 265 Street, Krakow, 30- 663 Poland; 2 Department of Pharmacology, Faculty of Medicine, Jagiellonian University Medical College, Grzegórzecka 16 Street, Krakow, 31-531 Poland; 3 Proteomics Laboratory, Centre for the Development of Therapies for Civilization and Age- Related Diseases CDT-CARD, Jagiellonian University Medical College, Skawińska 8 Street, Krakow, 31- 066 Poland; 4 Department of Pharmaceutical Technology and Biopharmaceutics, Faculty of Pharmacy, Jagiellonian University Medical College, Medyczna 9 Street, Krakow, 30-688 Poland

**Keywords:** Hypoxic-ischemic encephalopathy, Urine, Proteome, Neonate, Heme, Oxidative stress, Innate immunity, Inflammation

## Abstract

**Background:**

Neonatal hypoxic-ischemic encephalopathy (HIE) triggers systemic oxidative stress and redox imbalance, contributing to multi-organ injury. Urine is a noninvasive matrix for longitudinal profiling of molecular responses, yet time-resolved proteomic studies in HIE are limited.

**Methods:**

We performed longitudinal SWATH-MS proteomic profiling of urine from term neonates with moderate-to-severe HIE treated with therapeutic hypothermia (*n* = 16) and non-asphyxiated controls (*n* = 19) at six time points during the first eight days of life. Proteins identified in ≥ 80% of samples were quantified, and differential abundance, temporal clustering, pathway enrichment, and upstream regulatory networks were analyzed.

**Results:**

Approximately 1,000 proteins were quantified per sample. A total of 438 proteins were differentially abundant, with most changes transient and ten persistent. Early HIE urine showed marked elevation of hemoglobin subunits and sequential induction of haptoglobin and hemopexin, indicating staged heme scavenging and oxidative stress responses. Pathway enrichment revealed inhibition of neutrophil-associated innate immunity and activation of heme detoxification, lipoprotein remodeling, and PPAR signaling. Temporal clustering demonstrated stage-specific proteomic transitions, with partial normalization toward controls by days 6-8. Ingenuity Pathway Analysis identified six upstream regulators: Interleukin-1 alpha (IL1A), Tumor Necrosis Factor (TNF), ETS Homologous Factor (EHF), Peroxisome Proliferator-Activated Receptor Delta (PPARD), Thioredoxin-Interacting Protein (TXNIP), and Solute Carrier Family 2 Member 3 (SLC2A3), coordinating inflammation, redox control, and metabolic adaptation.

**Conclusions:**

We identified transient and sustained proteomic shifts that trace coordinated changes in oxidative stress, heme metabolism, and metabolic adaptation, alongside key regulators such as IL1A, TNF, EHF, PPARD, and TXNIP. The progression from early injury-related divergence to partial recovery by day 8 highlights the dynamic nature of post-insult remodeling. These findings support urinary proteomics as a robust, non-invasive tool for probing HIE pathophysiology and point to promising biomarker and pathway candidates for future studies.

**Supplementary Information:**

The online version contains supplementary material available at 10.1186/s40348-026-00222-7.

## Introduction

 Hypoxic-ischemic encephalopathy (HIE) is a major cause of neonatal brain injury and long-term neurodevelopmental disability [1, 2]. Beyond neuronal cell death, perinatal hypoxia-ischemia induces systemic oxidative stress through mitochondrial dysfunction, and generation of reactive oxygen species (ROS), overwhelming endogenous antioxidant, and detoxification mechanisms [3]. The resulting redox imbalance contributes to cellular injury in multiple organs and plays a key role in the pathophysiology of HIE [4]. Understanding the temporal dynamics of oxidative stress-related molecular responses may therefore inform the development of prognostic biomarkers and adjunctive therapeutic strategies.

Urine offers a uniquely non-invasive and clinically feasible matrix for longitudinal molecular profiling in neonates. Unlike blood or cerebrospinal fluid, repeated urine sampling poses minimal risk and can capture systemic responses to perinatal stress, including renal excretion of proteins linked to oxidative stress, ROS detoxification, immune modulation, and metabolic remodeling [5–8]. Previous studies have explored individual urinary biomarkers of kidney injury or inflammation, such as NGAL, KIM-1, or interleukin-18 [9], but proteome-wide analyses capturing time-resolved redox-related molecular signatures remain limited.

High-resolution, data-independent acquisition proteomics such as Sequential Windowed Acquisition of all Theoretical fragment ions (SWATH-MS) enables quantitative profiling of hundreds to thousands of proteins in complex biofluids, providing a systems-level view of molecular pathways perturbed during disease. Applying this approach to neonatal urine allows identification of proteins reflecting oxidative stress, heme handling, and metabolic regulation over the acute and subacute phases of HIE, which may remain undetectable using targeted or single-timepoint analyses.

In the present study, we performed longitudinal SWATH-MS proteomic profiling of urine from term neonates with HIE treated with therapeutic hypothermia and from non-asphyxiated term controls, sampled at six time points during the first eight days of life. We aimed to (1) characterize persistent and transient alterations in urinary proteins, (2) identify temporal trajectories of proteome perturbations, and (3) infer upstream regulatory networks underlying these dynamic molecular responses. By linking urinary proteomic changes to redox-sensitive pathways, this study provides novel insights into the systemic biology of neonatal HIE and highlights potential non-invasive markers for monitoring disease progression and recovery.

## Materials and methods

### Study groups

This single-center, prospective study was conducted in the Neonatal Intensive Care Unit (NICU) at the Institute of Pediatrics, Jagiellonian University Medical College, Cracow, Poland. Two cohorts of infants were consecutively and concurrently enrolled between April 2021 and July 2023:

1. Newborns with a gestational age (GA) ≥ 35 0/7 weeks who presented with moderate to severe hypoxic-ischemic encephalopathy (HIE), classified according to the Sarnat and Sarnat criteria, and qualified for therapeutic hypothermia. The inclusion criteria for the initiation of therapeutic hypothermia followed the protocol described by Shankaran et al. [10]. Whole-body cooling (to 33.5 °C) was initiated within 6 h after birth, maintained for 72 h, and followed by gradual rewarming over 6 h.
2. Term infants (GA ≥ 37 0/7 weeks) with mild postnatal adaptation problems, such as transient tachypnea of the newborn, admitted to the NICU within the first 24 hours of life.

Written informed consent was obtained from the parents or legal guardians prior to enrollment. Exclusion criteria for both cohorts were as follows: (1) major congenital anomalies of the heart or kidneys, or any structural abnormalities detected on cranial ultrasound upon admission; (2) multiple gestation; and (3) clinical suspicion of metabolic or genetic disorders. Demographic, perinatal, and clinical data, including details of the hospitalization course, were collected prospectively for all enrolled infants.

### Urine sampling and preparation, SWATH-MS quantitative proteomics, and data analysis

Urine sampling and preparation, SWATH-MS measurements and mass spectrometry data analysis was described in detail elsewhere [11, 12]. Briefly, urine samples for proteomic analysis were collected on days of life (DOL) 1, 2, 3, 4, 6, and 8. Samples were obtained noninvasively using sterile cotton balls placed in disposable diapers; urine was aspirated with a sterile syringe and transferred to sterile tubes. When urine was collected for clinical purposes using a sterile urine bag, an aliquot was taken for analysis. Samples contaminated with stool were discarded and recollected. Immediately after collection, urine samples were concentrated by centrifugation (10 min, 2600 × g, 4 °C; Vivaspin Turbo 4, 3 kDa MWCO, Sartorius, Germany) and stored at -80 °C until analysis. Undepleted urine samples were digested into peptides using the filter-aided sample preparation (FASP) protocol [13]. Samples (5 µg) were analyzed with TripleTOF 6600 + mass spectrometer (Sciex, Framingham, MA) operating in SWATH acquisition mode in the microflow chromatography regime, separated using a non-linear acetonitrile gradient. For SWATH acquisition, the spectra were collected in full scan mode (400–1250 Da), followed by one hundred SWATH MS/MS scans using a variable precursor isolation window approach, with m/z windows ranging from 6 to 90 Da. Acquired SWATH data were analyzed using project-specific spectral library in Spectronaut 19 (Biognosys, Schlieren, Switzerland). Data were filtered with 1% FDR at the peptide and protein level, while quantitation and interference correction were made at the MS2 level. The protein quantities were calculated by averaging the respective peptide intensities, while the latter were obtained as mean precursor quantities. To create a robust and reliable quantitative dataset, we selected only those proteins that were identified in at least 80% of the samples by at least two unique peptides and performed a missing value imputation using a global imputation strategy where the missing values were imputed on the basis of random sampling from a distribution of low-abundance signals taken throughout the entire experiment. The estimated significant absolute fold change cutoff was set at 2.0 to ensure the power of the statistical analysis. Data were normalized by global regression strategy, while statistical testing for differential protein abundance was performed using t-tests with multiple testing correction after Storey [14]. Functional grouping and pathway annotations were performed using ClueGO [15] under the Cytoscape 3.7.2 environment [16] with the use of PINE software [17]. The DP_GP_cluster repository (https://github.com/PrincetonUniversity/DP_GP_cluster), which is based on the Dirichlet process and Gaussian processes and is used for profile grouping [18], was utilized in the data clustering and visualization process. The script was adapted to the specifics of the analyzed dataset and executed in an environment compliant with the repository authors’ requirements (Python version 2.7). The alpha parameter, which controls the model’s tendency to create new clusters, was set to 0.5. Interpretation and selection of clusters for further detailed analysis was intended to identify proteins whose temporal changes were driving the observed convergence of expression profiles between the two examined cohorts at corresponding time points. An additional criterion for cluster selection was the number of proteins within each cluster that exhibited a high number of statistically significant changes (cut-off mean value of 2.0). Clusters considered less relevant for explaining the major temporal changes in urinary protein composition in the neonatal HIE group either did not exhibit similarity to the patterns observed in the correlation matrix or were characterized by a smaller number of proteins showing statistically significant differences in concentration during the observation period. Finally, we performed the analysis of the quantitative proteomic data using Ingenuity Pathway Analysis (Qiagen, Hilden, Germany) [19] using the set experimentally identified urine proteins in our SWATH dataset as reference proteome. A global heatmap was generated in Python (Pandas for data processing; Seaborn and Matplotlib for visualization), displaying each patient as an individual sample column and proteins as rows. Categories were indicated by bracket grouping without averaging or aggregation. The mass spectrometry proteomics data have been deposited to the ProteomeXchange Consortium via the PRIDE [20] partner repository with the dataset identifier PXD071111.

### Statistical analysis

Qualitative variables were analyzed using Fisher’s exact test, whereas continuous variables were compared with the Wilcoxon rank-sum test. Statistical significance was defined as a two-sided α level < 0.05. All statistical procedures were performed using JMP^®^ software, version 17.1.0 (JMP Statistical Discovery).

## Results

The studied groups did not differ in terms of gestational age, birth weight, sex, or mode of delivery. Infants in the HIE group were significantly more often their mothers’ first children and had significantly lower Apgar scores, pH, base excess, and lactate levels in the first postnatal measurements. Analysis of the hospital course showed that they required parenteral nutrition for a significantly longer period (Table 1). Among the children with HIE, 5 (31.25%) had moderate and 11 (68.75%) had severe HIE. Before initiation of therapeutic hypothermia, these infants were assessed using the Thompson score, with a mean of 6.23 (± 2.92) points. At the end of hospitalization, infants in the HIE group were assessed using the Test of Infant Motor Performance: 14 (87.5%) were classified as having average performance, while two (12.5%) were classified as having low-average performance.

**Table 1:** Demographic and hospitalization data of the study cohorts

Gestational age (weeks, mean (± SD))	Control group (*n* = 19)	HIE group (*n* = 16)	*p* value and test used
	38.53 (1.39)	38.69 (1.62)	0.7568^T^
Birth weight (g, mean (± SD))	3397 (555)	3426 (445)	0.8643^T^
Gender (# F/# total (% of F))	7/19 (36.84)	5/16 (31.25)	0.7284^P^
Pregnancy (median [Q1; Q3])	2 [1;4]	1 [1;1]	0.0138^W^
Delivery (median [Q1; Q3])	2 [1;3]	1 [1;1]	0.0153^W^
Cesarean section/vaginal delivery	11/8	9/7	0.9220^P^
1st min. Apgar score; median [Q1; Q3]	9 [7;10]	4 [2;6]	< 0.0001^W^
5th min. Apgar score; median [Q1; Q3]	10 [8;10]	7 [5;8]	< 0.0001^W^
10th min. Apgar score; median [Q1; Q3]	10 [9;10]	7 [7;8]	< 0.0001^W^
pH – arterial blood gases at 1 h of age, median [Q1; Q3]	7.28 [7.204;7.35]	7.01[6.9075;7.06]	< 0.0001^W^
BE – arterial blood gases at 1 h of age, mmol/l; median [Q1; Q3]	-5.1 [-5.3;-3.4]	-17.5 [-19.525;-14.625]	< 0.0001^W^
Lactate (blood) at 1 h of age, mmol/l; median [Q1; Q3]	3.275 [2.9425; 4.495]	9 [4.7;11.44]	< 0.0001^W^
Early-onset sepsis (*n*, (%))	1 (5.26)	0 (0.00)	0.3518^P^
Passive oxygen therapy (days, median [Q1; Q3])	0 [0;1]	0 [0;2]	0.6989^W^
CPAP/NIV (days, median [Q1; Q3])	1 [0;4]	2 [0;4]	0.2942
Mechanical ventilation (days, median [Q1; Q3])	0 [0;0]	0 [0;4.5]	0.0879^W^
Duration of parenteral nutrition (days, median [Q1; Q3])	2.5 [1;3.25]	6 [6;8]	< 0.0001^W^
Acute kidney injury, n (%)	0 (0.0)	0 (0.0)	-

^T^ - two sided t test; ^P^ - Pearson chi square test; ^W^ - Wilcoxon test

**Fig. 1:**
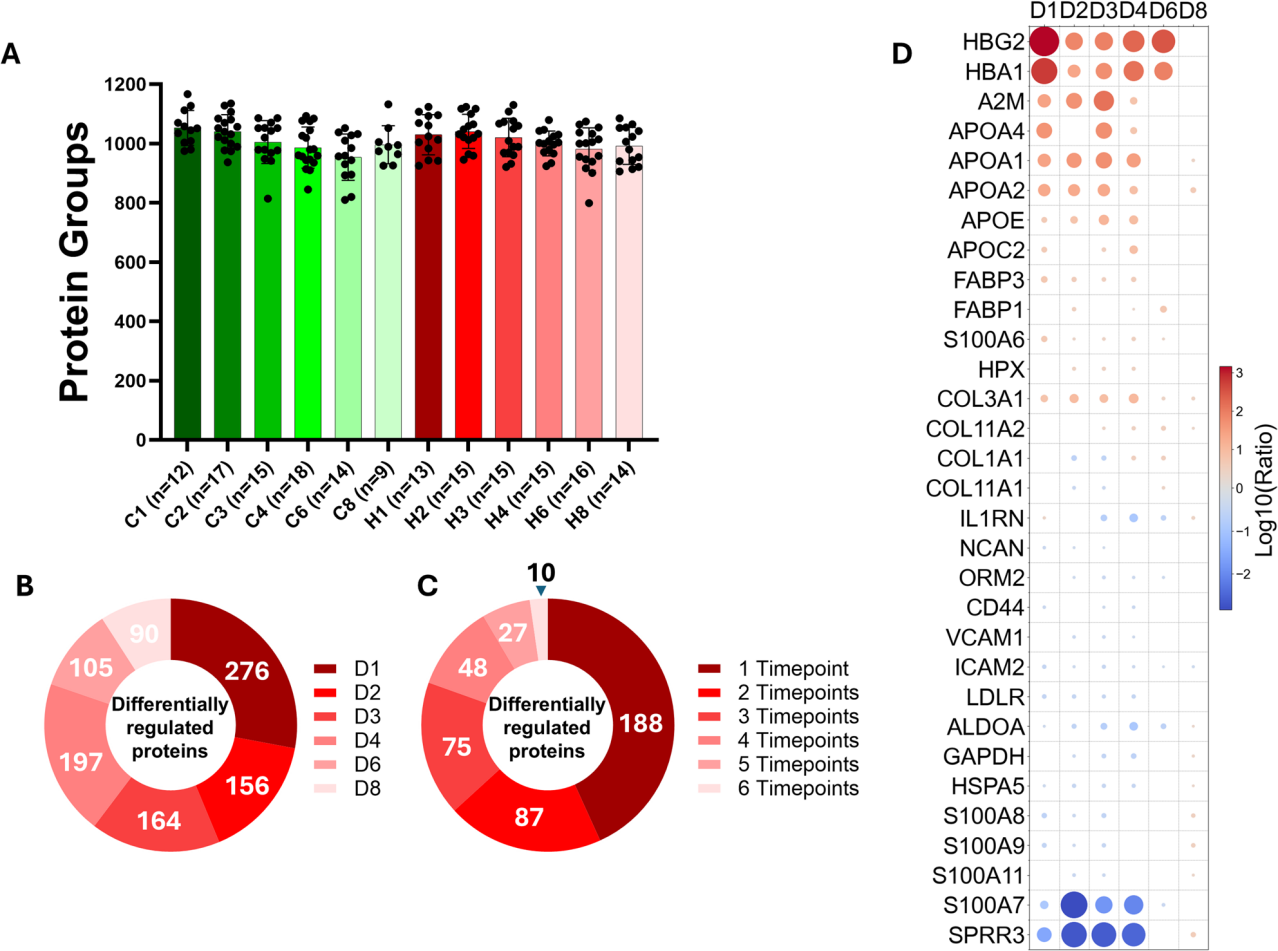
Overview of SWATH-MS quantification of the urinary proteome. Number of proteins identified per sample, showing a consistent average of ~ 1,000 proteins across all samples (**A**). Differentially regulated proteins grouped by time point. **B** Differentially regulated proteins classified by the number of time points at which they were significantly altered (**C**). Selected proteins exhibiting significant differences in urinary abundance between HIE and control groups (**D**)

On average, SWATH-MS analysis allowed the identification of approximately 1,000 proteins in each undepleted urine sample between both study cohorts and at all time points collected (Fig. 1A). As expected, urinary protein concentrations showed pronounced differences between groups, particularly during the first four days of observation. However, from day 6 onwards, the number of differentially abundant proteins decreased significantly by approximately two-fold compared to the initial observation phase (Fig. 1B). During the study period, 438 proteins were identified as differentially regulated, with the majority exhibiting significant alterations at a single time point. Notably, only ten proteins remained significantly altered throughout the entire study timeframe (Fig. 1C). Among the proteins most markedly altered in the HIE group compared to controls were hemoglobin, apolipoproteins, collagens, glycolytic enzymes, and members of the S100A protein family (Fig. 1D). Details of SWATH-MS-based urinary proteome quantitation are collected in Supplementary Tables S1-S6.

**Fig. 2:**
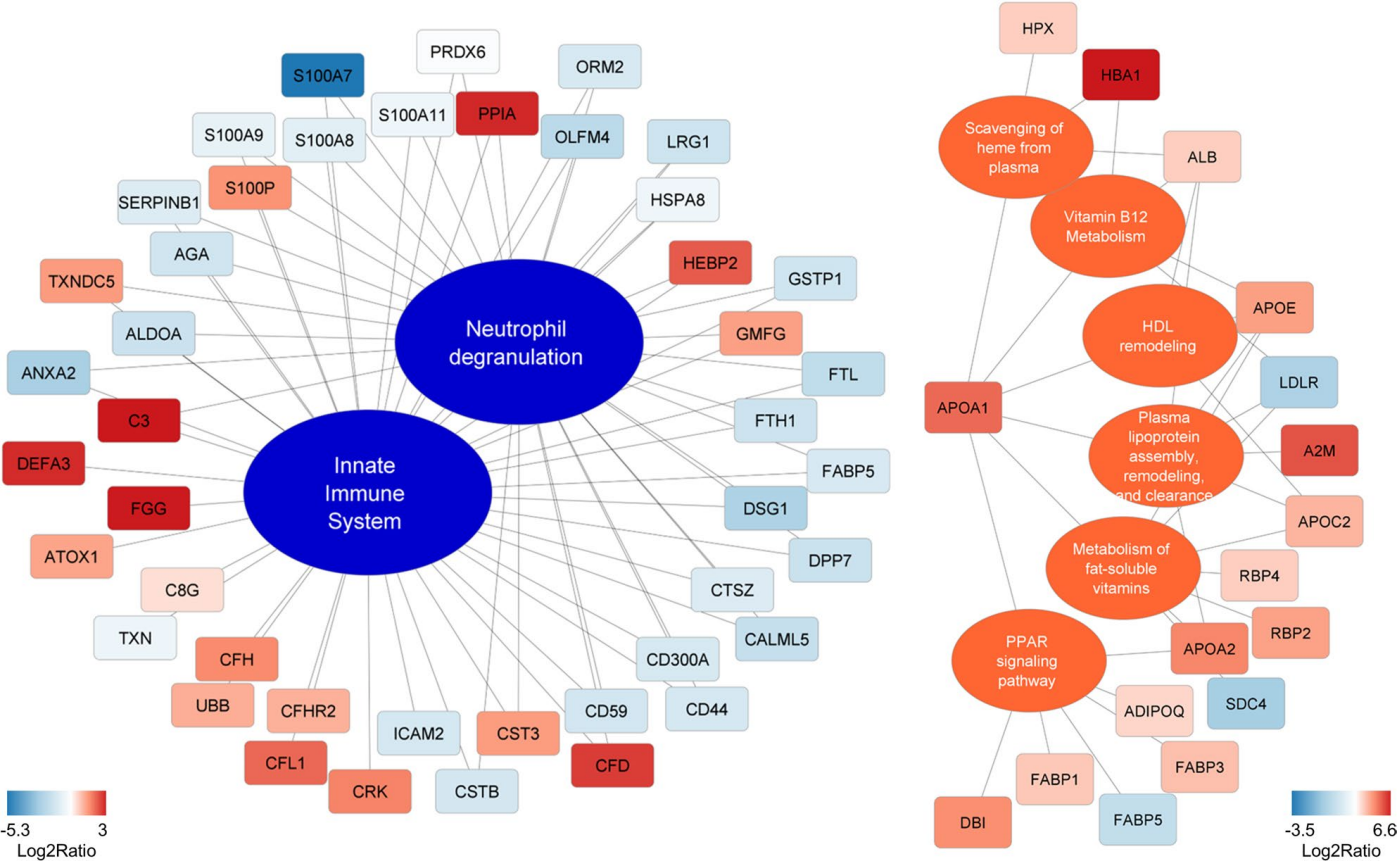
Pathway enrichment analysis based on time-resolved differences in the urinary proteome. Quantitative values (mean log₂ ratio) were calculated for proteins that were significantly different in abundance between HIE and control groups in at least 3 of the 6 time points

For the initial functional pathway analysis, we included only proteins that were significantly regulated at a minimum of three out of six time points (≥ 50%), thereby focusing on key and persistent urinary proteome alterations throughout the observation period. This analysis revealed an expected inhibition of innate immune system functions, associated with neutrophil activity, as inferred from protein abundance patterns. In contrast, activated pathways included those related to heme scavenging from plasma, lipoprotein assembly, and remodeling (primarily involving triglyceride- and HDL-associated particles) as well as peroxisome proliferator-activated receptor (PPAR) signaling (Fig. 2).

**Fig. 3:**
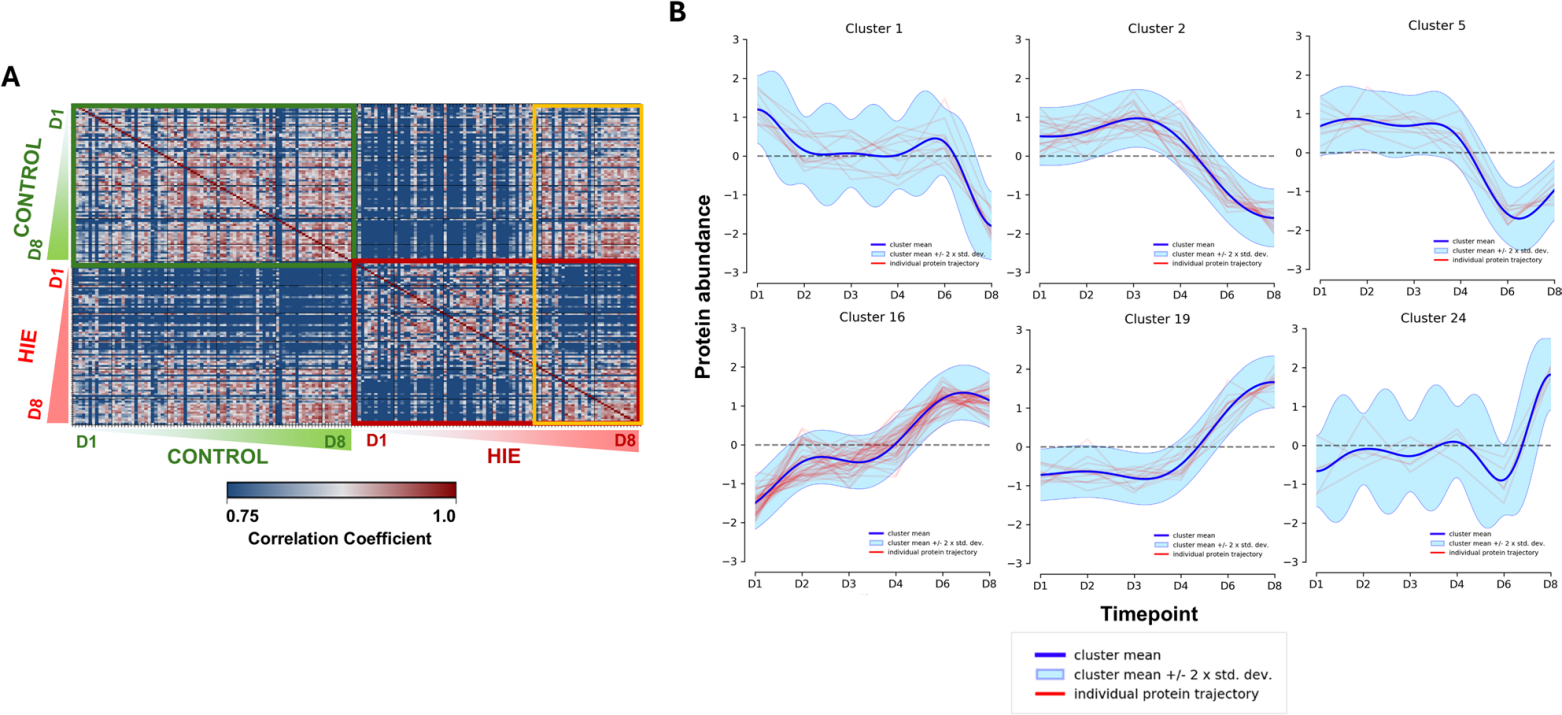
Time-resolved changes in the urinary proteome. Correlation matrix showing pairwise correlations of precursor quantities across all analyzed samples. Rows and columns are ordered by condition, time point, and replicate. The shift in correlation structure observed on days 6 and 8 is highlighted in yellow. Green and pink wedges (representing the control and HIE groups, respectively) indicate increasing durations of the observation period. **A** Trajectory-based clusters of similarly regulated proteins whose quantitative profiles are consistent with the correlation patterns. Protein abundances were derived by mean-centering the log₁₀ ratio values followed by standardization (scaling by standard deviation) (**B**)

Considering the distinct quantitative profiles of the regulated proteins between the groups during the observation period, we performed a detailed analysis of the dynamics of time-resolved proteome perturbations. The correlation matrix of all SWATH-MS measured samples demonstrated that during the first four days, differences between the HIE and control groups were more pronounced, while intra-group similarities among HIE newborns were highest. These similarities diminished from day 6 onwards, with the urinary proteome composition of the HIE group progressively converging toward that of controls (Fig. 3A). To identify proteins underlying these changes, we conducted a cluster analysis of their quantitative trajectories, classifying the 438 regulated proteins into 26 clusters (Suppl. Fig. S1, Suppl. Tab. S7). From these, six clusters exhibiting temporal patterns consistent with the correlation matrix (quantitative changes on days 6 and 8) were selected for further examination (Fig. 3B). The proteins within these clusters were functionally linked to most of the processes indicated by pathway analysis (Suppl. Fig. S2-S4). Moreover, based on the obtained clusters, the time-resolved interactions of hemoglobin and proteins responsible for its plasma turnover can be deciphered. Hemoglobin chains were markedly upregulated in urine samples from newborns with HIE, remaining elevated until day 6. This trend was associated with changes in hemoglobin-binding haptoglobin (HP), as hemoglobin levels normalized in a timely-manner after HP induction. Additionally, hemoglobin turnover seems to be further supported by hemopexin (HPX) prior to HP upregulation, with HPX showing a nearly three-fold increase from days 2 to 4, potentially constituting a protective mechanism against free heme toxicity (Suppl. Fig. S2, Suppl. Tab. S7). Supplementary Table S5 presents a global heatmap summarizing the quantification data for all included patients.

### Upstream regulators and integrated molecular networks

**Fig. 4:**
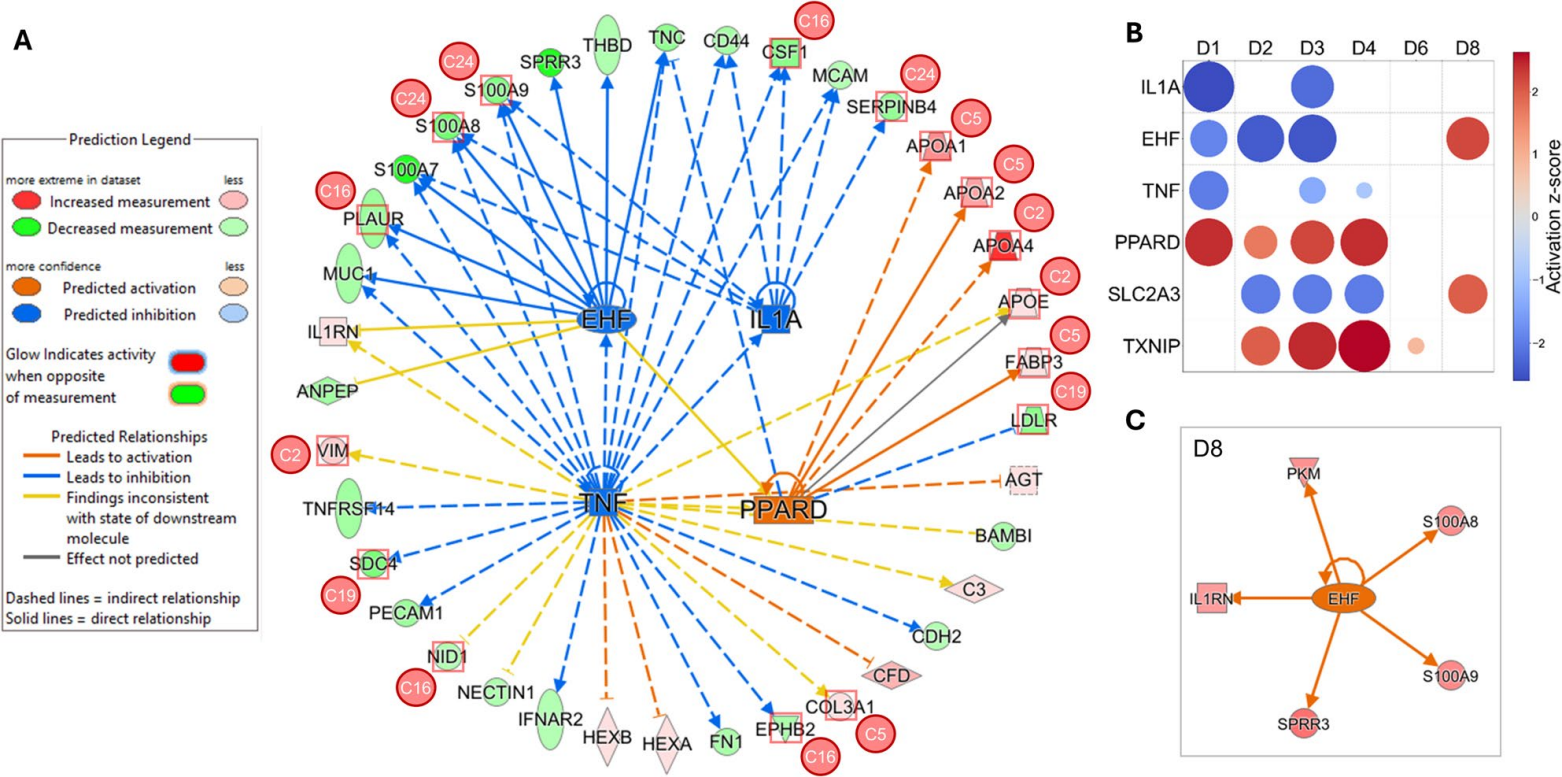
Upstream regulators inferred from time-resolved urinary proteome dynamics. Network of regulated urinary proteins identified on day 1, overlaid with the inferred activity states of upstream regulators and their assignment to temporal trajectory clusters (**A**). Interconnected regulatory relationships among upstream regulators across the full observation period (**B**). Protein interaction network centered on EHF on day 8 illustrating the inferred reversal of its early activity state (**C**)

Finally, we applied Ingenuity Pathway Analysis (IPA, Qiagen) to identify key upstream regulators underlying the differences observed in urinary proteomes of HIE neonates. A unique feature of IPA is its ability to predict upstream regulators based on the measured differential abundance of downstream proteins, thereby enabling the construction of regulatory networks contextualizing the experimental proteomic findings (Fig. 4A). This analysis identified six upstream regulators that were significantly and repeatedly modulated throughout the study period: interleukin-1 alpha (IL1A), tumor necrosis factor (TNF), ETS homologous factor (EHF), peroxisome proliferator-activated receptor delta (PPARD), thioredoxin-interacting protein (TXNIP), and solute carrier family 2 member 3 (SLC2A3) (Fig. 4B). Their regulation was temporally variable and interdependent. For example, the pro-inflammatory cytokines IL1A and TNF were predicted to be inhibited during the first four days, coinciding with the repression of EHF activity. However, EHF showed activation by the eighth day. Similarly, SLC2A3 (GLUT3) showed a temporal shift from inhibition to activation. PPARD and TXNIP emerged as functionally related upstream regulators (Fig. 4B). Importantly, multiple proteins from which the activity of the upstream regulators was inferred were localized within the six clusters whose quantitative trajectories mirrored the major proteome dynamics identified by SWATH-MS (Fig. 3B).

## Discussion

In this longitudinal study, we characterized dynamic alterations in the urinary proteome of term neonates with HIE treated with therapeutic hypothermia and compared them to term non-asphyxiated controls across the first eight days of life. Obtained findings demonstrate that the urinary proteome exhibits highly dynamic, injury-related molecular alterations, particularly during the first four postnatal days, with a partial convergence toward control profiles by days 6 and 8.

Our results indicate that neonatal urine is a sensitive, non-invasive window into systemic oxidative stress, heme turnover, and redox-regulated pathways triggered by perinatal hypoxia-ischemia.

### Early post-hypoxic phase - multifaceted proteome perturbation with prominent heme turnover and oxidative stress signatures

The pronounced proteomic divergence between HIE and control newborns during the first four days of life is consistent with the acute pathophysiological cascade triggered by perinatal hypoxia-ischemia. The identification of more than 400 differentially abundant proteins, yet only a small subset with persistent alterations, indicates that the urinary proteome in HIE is dominated by rapid, injury-driven fluctuations rather than stable long-term changes. This pattern parallels prior observations of highly dynamic systemic metabolic, inflammatory, and oxidative responses during and shortly after therapeutic hypothermia [21].

Among the earliest and most striking alterations was the increased urinary excretion of hemoglobin subunits and related proteins. Hemoglobinuria in this context may reflect hemolysis, tissue breakdown, renal tubular stress, or transient changes in glomerular permeability associated with perinatal asphyxia [22, 23]. The elevated hemoglobin subunits, together with the sequential induction of haptoglobin (HP) and hemopexin (HPX), point to early activation of heme-scavenging and detoxification pathways. Free hemoglobin and heme are potent pro-oxidants [24] capable of generating reactive oxygen species and exacerbating tissue injury. The temporal pattern observed - HPX induction preceding subsequent HP upregulation - suggests a staged protective response to buffer free heme toxicity. HPX, with its high-affinity heme binding, provides immediate neutralization of circulating heme, whereas HP induction may support longer-term clearance of hemoglobin complexes [25]. Persistence of these changes into the early postnatal period underscores the sustained oxidative challenge following HIE. These findings further suggest that urinary HP and HPX may represent noninvasive biomarkers of systemic heme turnover and oxidative stress dynamics in affected newborns. Interestingly, Zhu et al., in their iTRAQ proteomic serum analysis, found a significant upregulation of haptoglobin in blood samples from neonates with HIE and identified haptoglobin as a promising marker of HIE [26].

Additional differentially abundant proteins, including apolipoproteins, collagens, glycolytic enzymes, and S100A family members, highlight broad systemic perturbations involving lipid metabolism, extracellular matrix remodeling, energy homeostasis, and inflammation. Their integration within the time-resolved clusters and pathway networks supports the interpretation that these changes reflect coordinated responses to hypoxia-induced oxidative stress rather than isolated molecular events.

### Functional pathway changes and inferred biology

Pathway enrichment of proteins significantly altered at ≥ 3 time points revealed early inhibition of neutrophil-associated innate immune functions, accompanied by activation of heme detoxification, lipoprotein assembly and remodeling, and peroxisome proliferator-activated receptor (PPAR) signaling. Suppression of neutrophil-related pathways in the acute phase may reflect a transient compensatory response aimed at limiting inflammation-driven oxidative damage - a phenomenon described in patients with ischemic stroke as an adaptive mechanism to mitigate secondary injury [27]. Notably, early downregulation of neutrophil pathways is also consistent with the well-documented immunosuppression observed in critically ill patients [28], who often exhibit reduced neutrophil recruitment and functional exhaustion during severe systemic stress. Activation of PPAR pathways, particularly PPARD, is consistent with shifts in lipid oxidation, mitochondrial regulation, and metabolic reprogramming, all of which are central to cellular resilience under hypoxic and oxidative stress [29, 30]. The observed modulation of lipoprotein remodeling, including pathways associated with HDL and triglyceride-rich particles, aligns with emerging evidence that lipid transport and lipoprotein-associated antioxidants play important roles in neonatal stress physiology [31]. Collectively, these pathway-level changes indicate that urinary proteomics captures not only redox disturbances but also the coordinated interplay between metabolic adaptation, immune modulation, and oxidative damage mitigation following hypoxic-ischemic injury.

### Temporal clustering reveals stage-specific proteomic transitions

Trajectory-based clustering of the 438 regulated proteins identified six clusters whose quantitative patterns closely mirrored the temporal shift observed in the correlation matrix-marked divergence between HIE and control infants during days 1–4, followed by partial convergence on days 6–8. Proteins within these clusters were functionally linked to the major pathways highlighted by enrichment analysis, indicating that the urinary proteome captures coordinated, stage-specific biological responses rather than isolated fluctuations in individual proteins. The progressive alignment of proteomic profiles with controls after day 6 may reflect partial systemic recovery, attenuation of early oxidative and inflammatory disturbances, or reduced renal elimination of injury-associated proteins as homeostasis is gradually restored. Notably, this temporal pattern aligns with the expected clinical trajectory in cooled neonates, in whom the most pronounced metabolic, inflammatory, and oxidative stress processes occur within the first 72 h, followed by stabilization during the rewarming and early recovery phases [32–34].

### Upstream regulators and integrated molecular networks

Ingenuity Pathway Analysis identified six upstream regulators: IL1A, TNF, EHF, PPARD, TXNIP, and SLC2A3, whose predicted activity patterns closely paralleled the major temporal trajectories of the urinary proteome. Among these, EHF is known as a key epithelial transcription factor involved in cytokine-driven inflammatory responses, epithelial integrity, and wound repair [35]. Its expression is regulated by pro-inflammatory cytokines, including IL1A and TNF, through NF-κB-dependent signaling [36] Experimental studies have shown that EHF depletion impairs cytokine production required for neutrophil recruitment and delays epithelial restitution, underscoring its importance for coordinated inflammatory and repair processes [35]. Although EHF biology has been explored primarily in pulmonary and gastrointestinal epithelia [37] systemic inflammatory crosstalk following neonatal hypoxic-ischemic injury could plausibly modulate its activity in extra-cerebral tissues.

In the present study, predicted early inhibition of IL1A and TNF was accompanied by parallel repression of EHF during the first four days, aligning with a transient dampening of cytokine-driven epithelial and immune responses under acute oxidative stress. By day 8, EHF was predicted to shift toward activation, potentially reflecting partial restoration of cytokine signaling and re-engagement of epithelial repair pathways. This temporal pattern supports the broader concept that urinary proteomics captures systemic inflammatory and redox-regulated adaptations extending beyond the central nervous system in neonatal HIE.

PPARD and TXNIP displayed tightly synchronized predicted activities during the early postnatal period, suggesting coordinated regulation of redox and metabolic pathways. PPARD is increasingly recognized as a central modulator of neuroinflammation and cellular resilience in ischemic and traumatic neural injury [38]. Experimental studies indicate that PPARD activation can attenuate neuronal apoptosis and oxidative damage, in part through transcriptional programs intersecting with TXNIP-mediated redox control [39]. TXNIP itself serves as a critical regulator of oxidative stress and inflammasome activation, acting both as an inhibitor of thioredoxin and an amplifier of ROS-dependent injury [40]. Their synchronous behavior in our dataset therefore points to an interplay between PPARD signaling and TXNIP-linked oxidative pathways during the systemic response to hypoxic-ischemic injury.

SLC2A3 (GLUT3), a high-affinity neuronal glucose transporter [41], also demonstrated a transition from predicted inhibition to activation, potentially reflecting metabolic reprogramming in response to evolving energy and redox demands during recovery.

Collectively, these upstream regulators delineate a dynamic molecular network linking inflammatory modulation, oxidative stress regulation, and metabolic adaptation throughout the post-injury period in neonatal HIE. Integration of cluster analysis with upstream regulator analysis validated the most relevant temporal changes in the urinary proteome relative to predicted alterations in upstream regulator activity and revealed that proteins involved in modulation of this activity exhibited the largest changes in urinary concentration over the observation period.

### Clinical and translational implications

Our findings highlight the promise of urinary proteomics as a noninvasive approach for monitoring oxidative stress-linked systemic responses in neonates with HIE. Proteins associated with heme detoxification, redox regulation, and metabolic adaptation emerge as potential biomarkers for early injury identification, longitudinal disease monitoring, and risk stratification. Importantly, the time-resolved trajectories described here provide a framework for defining stage-specific biomarker windows, which may outperform single-timepoint measurements and support more precise clinical decision-making. Beyond biomarker discovery, these results advance understanding of the systemic molecular adaptations to neonatal HIE and may guide the development of targeted neuroprotective or anti-oxidative interventions.

### Limitations

This study has several limitations. Most notably, the absence of a non-cooled HIE control group precludes distinguishing proteomic changes attributable to hypoxic-ischemic injury from those influenced by therapeutic hypothermia. However, at present, therapeutic hypothermia remains the standard of care for infants with moderate to severe perinatal asphyxia. The absence of a non-cooled HIE control group precludes direct disentanglement of proteomic changes driven by hypoxic-ischemic injury itself from those modulated by therapeutic hypothermia. As a result, causal attribution of specific proteomic trajectories to injury versus treatment effects is limited, and the identified signatures should be interpreted as reflecting the combined biological response to HIE under current standard-of-care conditions. Moreover, although disease severity is a key determinant of outcome in hypoxic-ischemic encephalopathy, the current study was underpowered to assess severity-specific proteomic patterns. Future studies with larger, severity-balanced cohorts will be required to disentangle shared versus severity-dependent molecular responses. Additionally, urinary proteomic signatures reflect systemic and renal physiology and cannot be assumed to directly mirror cerebral injury mechanisms without complementary tissue or circulating biomarker data. The modest sample size further limits generalizability, underscoring the need for validation in larger, independent cohorts and integration with clinical outcomes such as MRI injury scores or long-term neurodevelopment. Despite these constraints, the temporal consistency and biological plausibility of the identified pathways support the robustness of our findings.

## Conclusions

In conclusion, by integrating quantitative SWATH-MS profiling with temporal clustering and upstream regulator modeling, this study delivers a systems-level view of the molecular responses to neonatal hypoxic-ischemic injury. Our analysis fulfilled the study aims by identifying both transient and persistent proteomic alterations, revealing coordinated trajectories of oxidative stress, heme turnover, and metabolic remodeling, and uncovering a set of regulators, including IL1A, TNF, EHF, PPARD, and TXNIP, that shape these responses over time. The shift from early, injury-driven proteome divergence to partial normalization by day 8 underscores the dynamic interplay between injury progression and recovery. Together, these findings position urinary proteomics as a powerful, non-invasive approach for elucidating complex pathophysiological processes in HIE and highlight promising candidate biomarkers and pathways for future mechanistic and translational research.

## Supplementary Information

Supplementary Material 1.

Supplementary Material 2.

Supplementary Material 3.

Supplementary Material 4.

Supplementary Material 5.

Supplementary Material 6.

Supplementary Material 7.

## Data Availability

All the data analyzed during this study are included in this article. Further inquiries can be directed to the corresponding author.
