# Supplementary figures and images for: Time-resolved urinary proteomics reveals heme-associated oxidative stress responses in neonatal hypoxic-ischaemic encephalopathy
Source: Mol Cell Pediatr. 2026 Mar 4;13:10. doi: 10.1186/s40348-026-00222-7 (PMC12963591; doi:10.1186/s40348-026-00222-7)

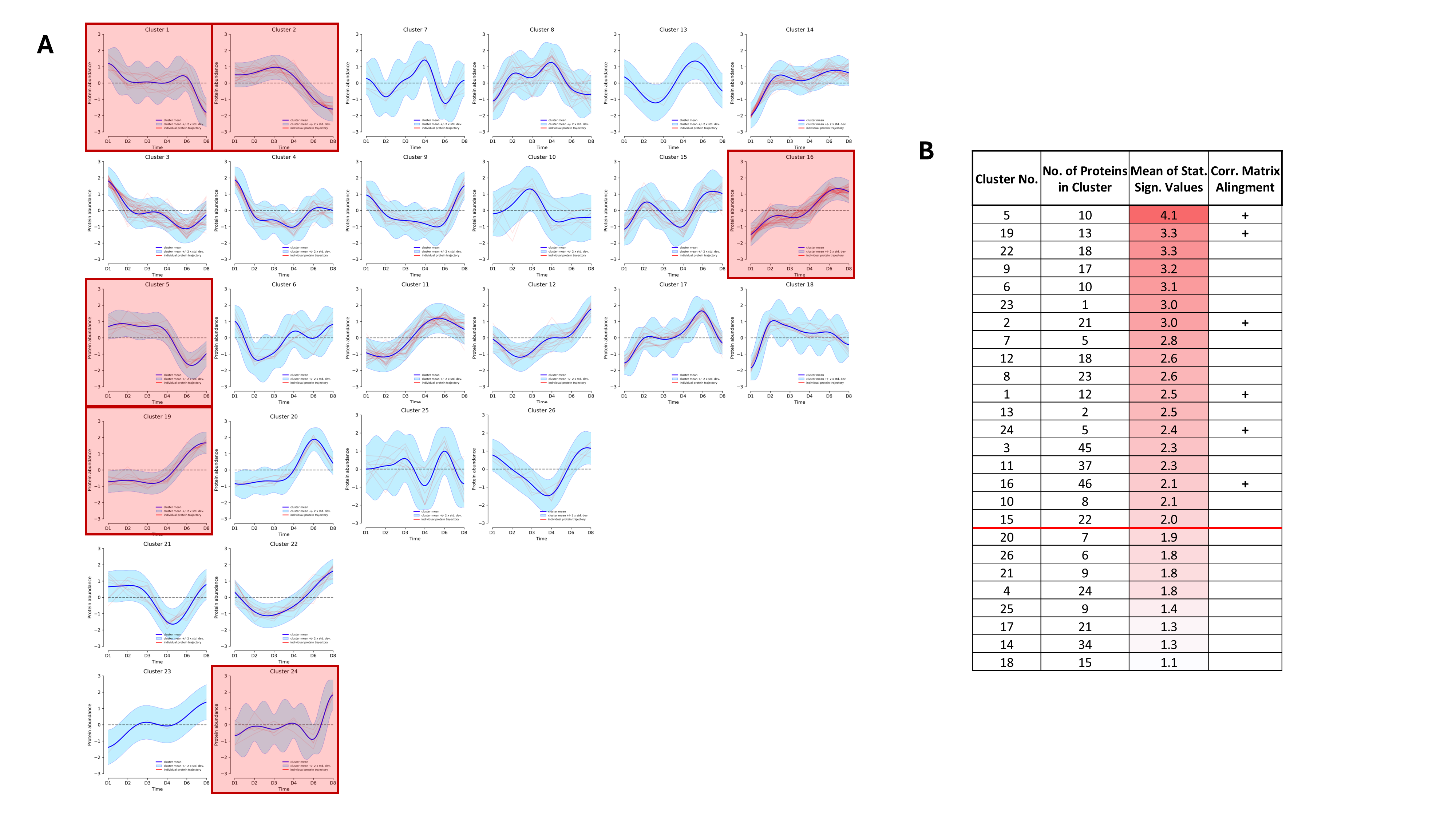

Supplement: Supplementary file 1 — Supplementary Material 1. [file 40348_2026_222_MOESM1_ESM.tif]

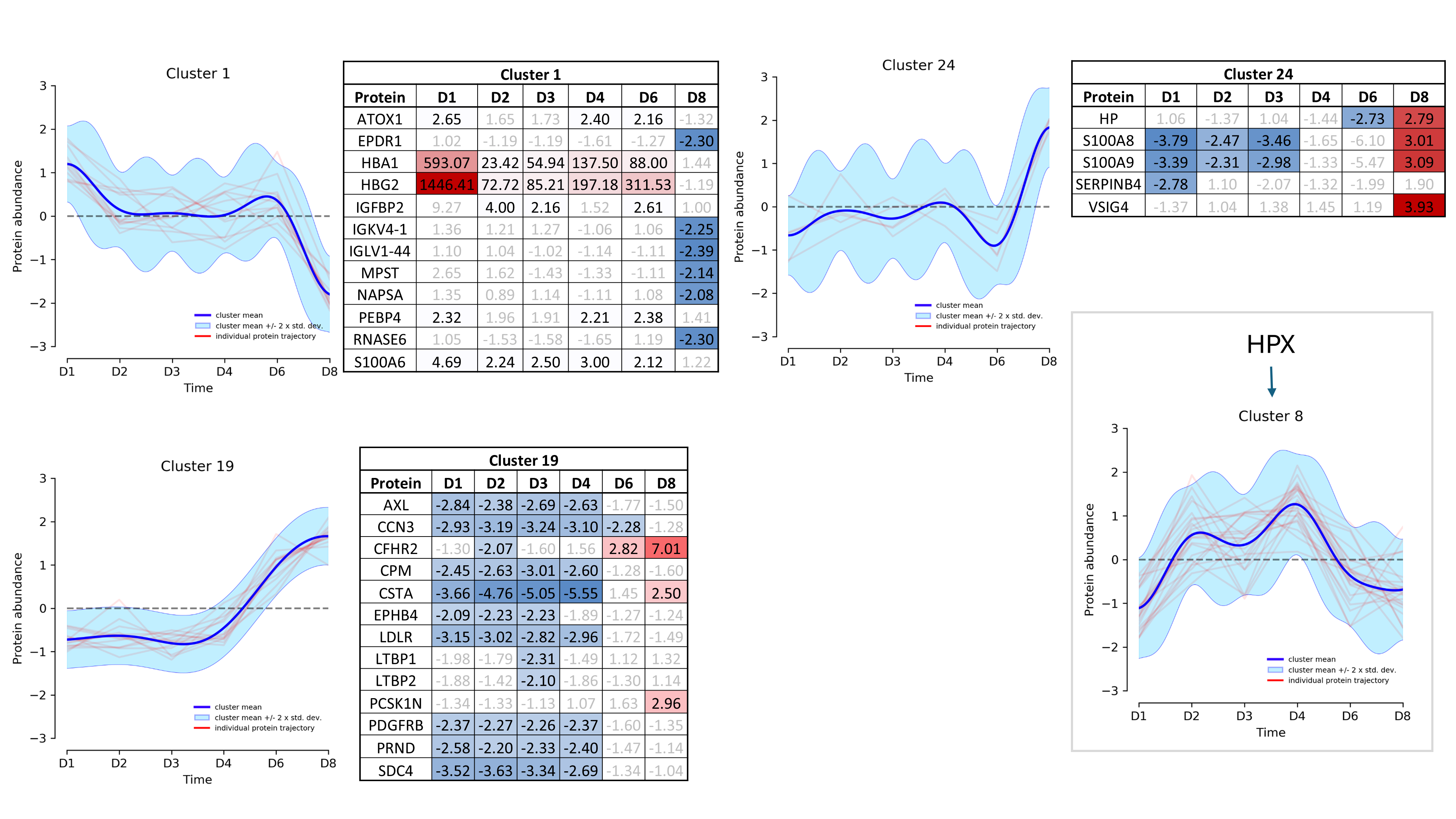

Supplement: Supplementary file 4 — Supplementary Material 4. [file 40348_2026_222_MOESM4_ESM.tif]

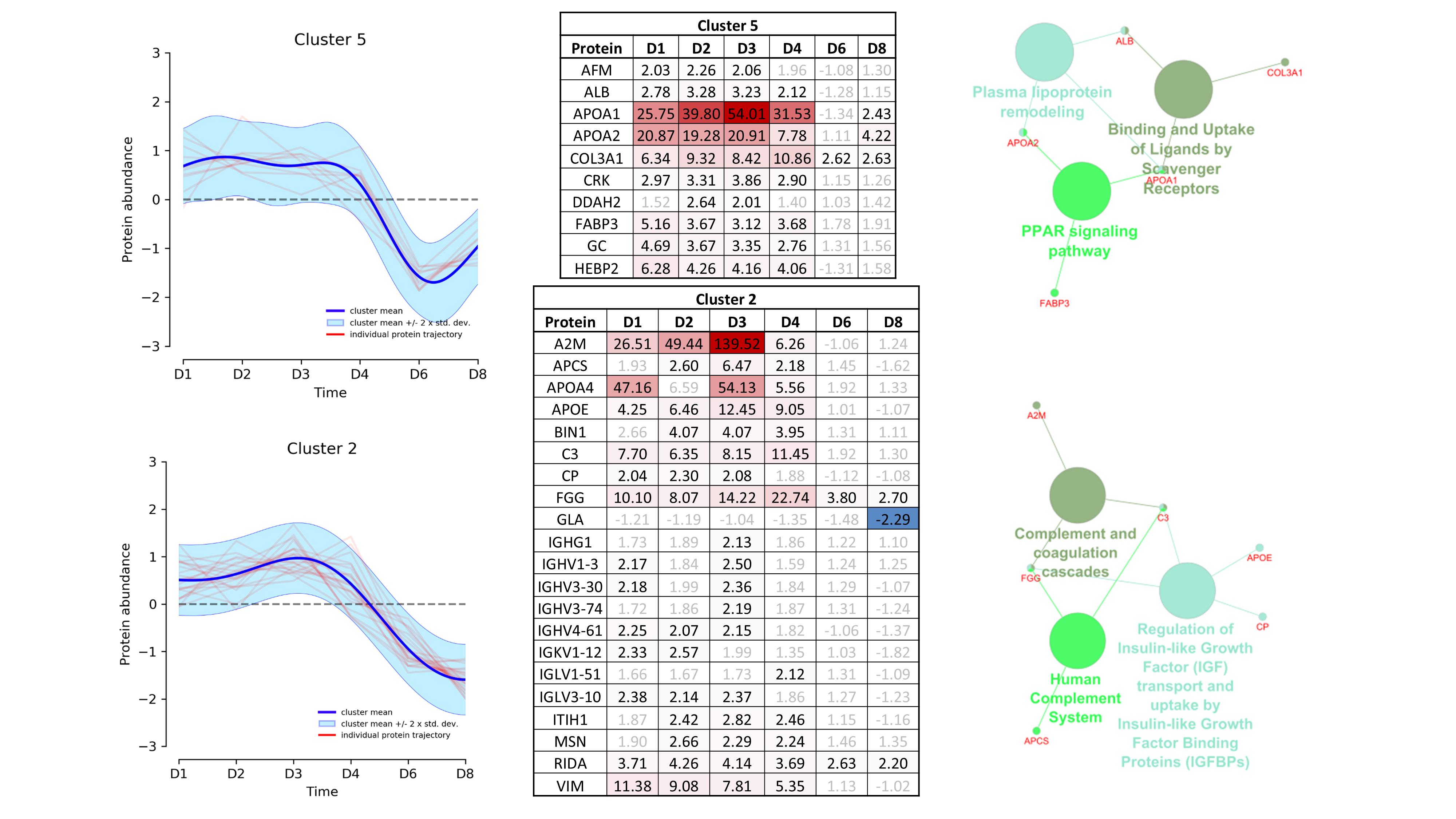

Supplement: Supplementary file 5 — Supplementary Material 5. [file 40348_2026_222_MOESM5_ESM.tif]

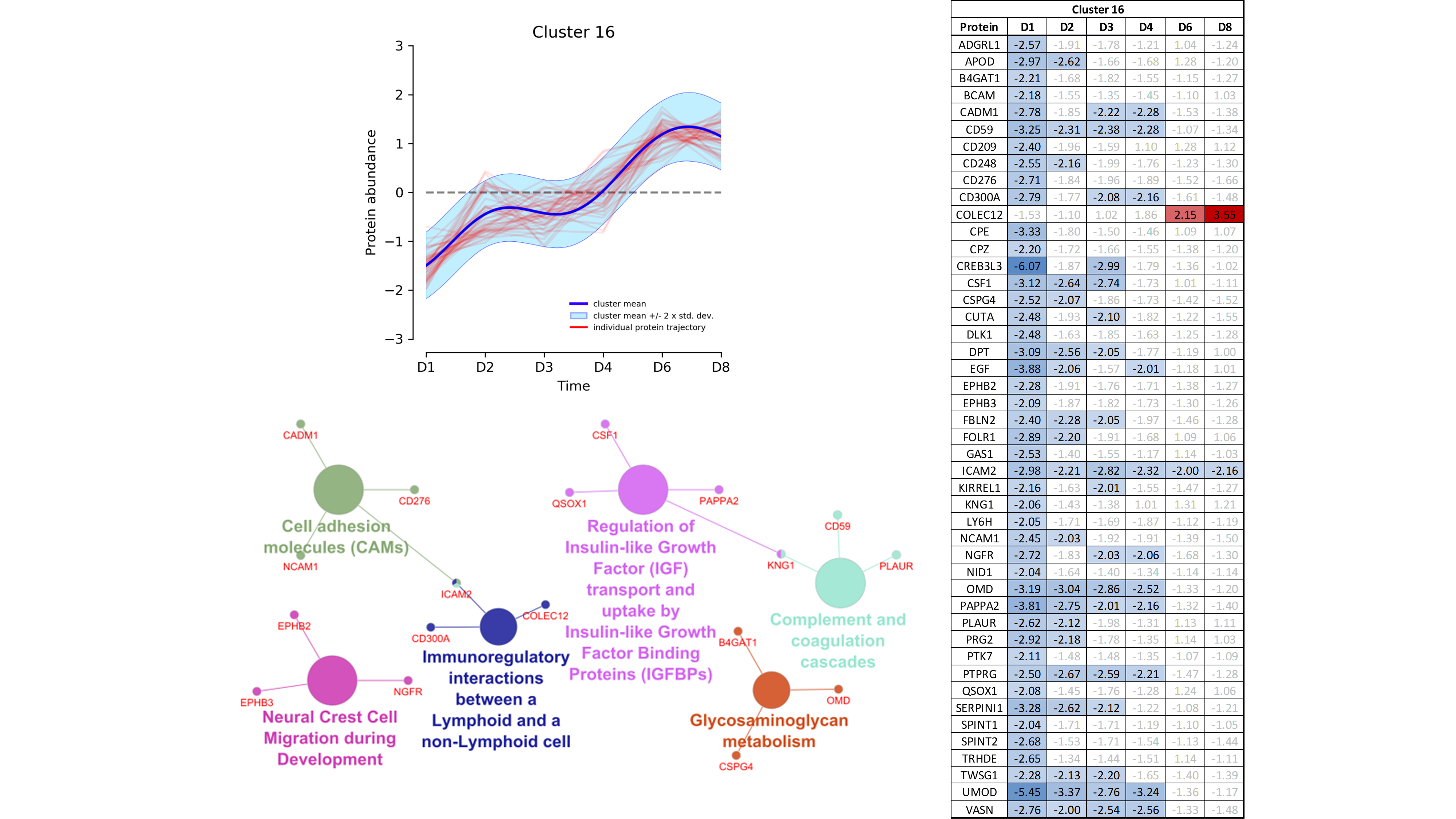

Supplement: Supplementary file 6 — Supplementary Material 6. [file 40348_2026_222_MOESM6_ESM.tif]

Global heatmap

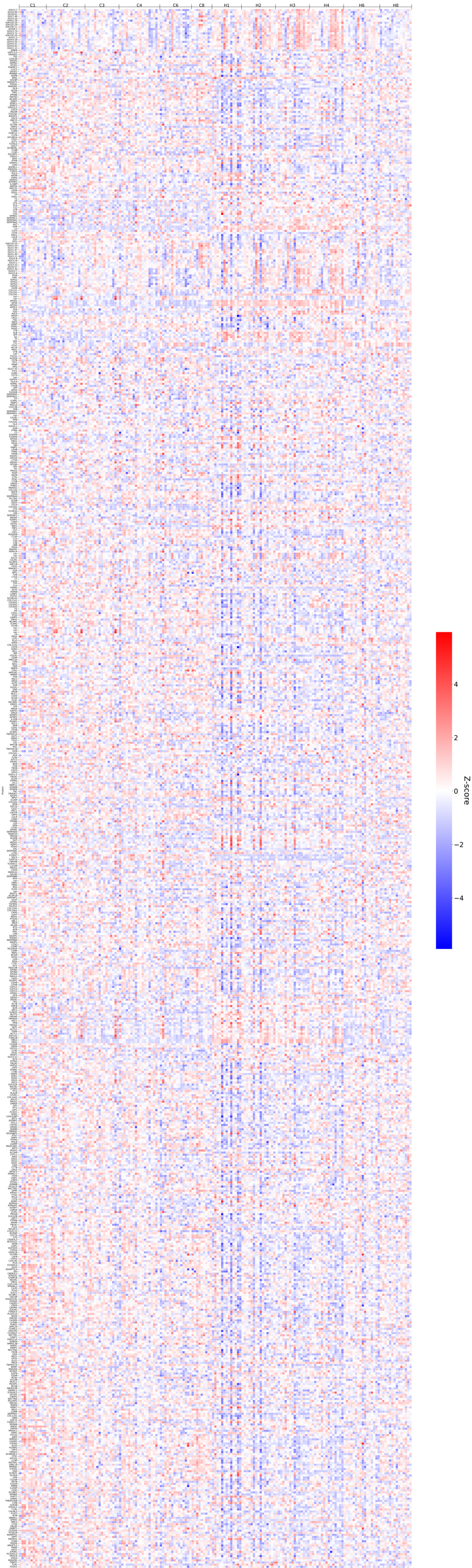

Supplement: Supplementary file 7 — Supplementary Material 7. [file 40348_2026_222_MOESM7_ESM.pdf]
